# Regeneration in Spinal Disease: Therapeutic Role of Hypoxia-Inducible Factor-1 Alpha in Regeneration of Degenerative Intervertebral Disc

**DOI:** 10.3390/ijms22105281

**Published:** 2021-05-17

**Authors:** Jin-Woo Kim, Neunghan Jeon, Dong-Eun Shin, So-Young Lee, Myongwhan Kim, Dong Hun Han, Jae Yeon Shin, Soonchul Lee

**Affiliations:** 1 Department of Orthopaedic Surgery, Nowon Eulji Medical Center, Eulji University, Seoul 01830, Korea; jinwu3911@hanmail.net (J.-W.K.); bless2dness@hanmail.net (N.J.); billy0327@daum.net (M.K.); 2 Department of Orthopaedic Surgery, CHA Bundang Medical Center, CHA University, Seongnam-si 13488, Korea; shinde@cha.ac.kr (D.-E.S.); hdh7179@gmail.com (D.H.H.); 3 Department of Internal Medicine, CHA Bundang Medical Center, CHA University School of Medicine, Seongnam-si 13488, Korea; ysy0119@cha.ac.kr; 4 Department of Computer Science, College of IT Engineering, SeMyung University, Jechun 27136, Korea; zhyeon838@gmail.com

**Keywords:** hypoxia-inducible factor-1α, regeneration, intervertebral disc, disc degeneration, nucleus pulposus

## Abstract

The intervertebral disc (IVD) is a complex joint structure comprising three primary components—namely, nucleus pulposus (NP), annulus fibrosus (AF), and cartilaginous endplate (CEP). The IVD retrieves oxygen from the surrounding vertebral body through CEP by diffusion and likely generates ATP via anaerobic glycolysis. IVD degeneration is characterized by a cascade of cellular, compositional, structural changes. With advanced age, pronounced changes occur in the composition of the disc extracellular matrix (ECM). NP and AF cells in the IVD possess poor regenerative capacity compared with that of other tissues. Hypoxia-inducible factor (HIF) is a master transcription factor that initiates a coordinated cellular cascade in response to a low oxygen tension environment, including the regulation of numerous enzymes in response to hypoxia. HIF-1α is essential for NP development and homeostasis and is involved in various processes of IVD degeneration process, promotes ECM in NP, maintains the metabolic activities of NP, and regulates dystrophic mineralization of NP, as well as angiogenesis, autophagy, and apoptosis during IVD degeneration. HIF-1α may, therefore, represent a diagnostic tool for early IVD degeneration and a therapeutic target for inhibiting IVD degeneration

## 1. Introduction

The intervertebral disc (IVD) links adjacent vertebral bodies and protect against damage during extremes physical loads. IVD comprises a central gelatinous nucleus pulposus (NP) surrounded by a fibrous annulus fibrosus (AF) and is joined to adjacent vertebrae by cartilaginous endplate (CEP) [1,2,3] NP and AF can withstand normal activities; NP can resist axial compression while AF can endure tensile stress [4]; they are supplied oxygen via diffusion through the CEP.

IVD regulates homeostasis by actively maintaining a balance between the anabolic and catabolic metabolism of IVD cells. IVD retrieves oxygen from the surrounding vertebral body through CEP via diffusion and may generate adenosine triphosphate (ATP) using anaerobic glycolysis [5,6]. The aggrecan-rich NP is an avascular tissue that is sparsely populated with NP cells in a hypoxic environment [7]. IVD is the largest avascular structure in the human body [1,8]; few blood vessels infiltrate the superficial region of the CEP and the outer third of the AF; however, none of these vessels infiltrate the NP [9,10].

IVD degeneration is characterized by a cascade of cellular, compositional, structural, and functional changes [11,12]. IVD degeneration is a major cause of spinal disorders associated with low back pain, which is influenced by several factors and has high morbidity and mortality [11,13,14].

Oxygen reaches the NP predominantly through diffusion, thereby imposing a hypoxic state on the NP cells [15,16], which is in turn enhanced by the loss of CEP permeability during IVD degeneration [17]. Hypoxia is an important cellular stress mechanism with significant pathological implications in numerous diseases, such as cerebral ischemia, cancer, and chronic degenerative disorders [18,19].

Hypoxia-inducible factor (HIF) is a master transcription factor that initiates a coordinated cellular cascade in response to a low oxygen tension environment, including the regulation of numerous enzymes in response to hypoxia [19,20,21,22,23,24,25,26]. HIF has been shown to play an essential role in cellular and systemic homeostatic response to hypoxia. Recently, HIF expression in NP cells was reported by many groups [9,17,23,24,27,28,29,30,31,32,33]. Moreover, HIF-1α is essential for NP development and homeostasis and could be involved in IVD degeneration in humans [34,35].

This review will summarize structure, function, and pathogenesis of IVD. Furthermore, we discuss the regulatory roles of HIF-1α in the biological behaviors of IVD, current data on the expression of HIF-1α in IVD, and roles of HIF-1α plays in the regulation of the phenotypes, survival, metabolism, regulation of extra cellular matrix, and dystrophic mineralization of NP cells of IVD.

## 2. Structure and Function of the IVD

The anatomical function of the IVD is to stabilize the spine by anchoring adjacent vertebral bodies and to enable flexible movement of the spine, while its physiological function is to absorb shock and evenly distribute axial pressure [36].

**Figure 1:**
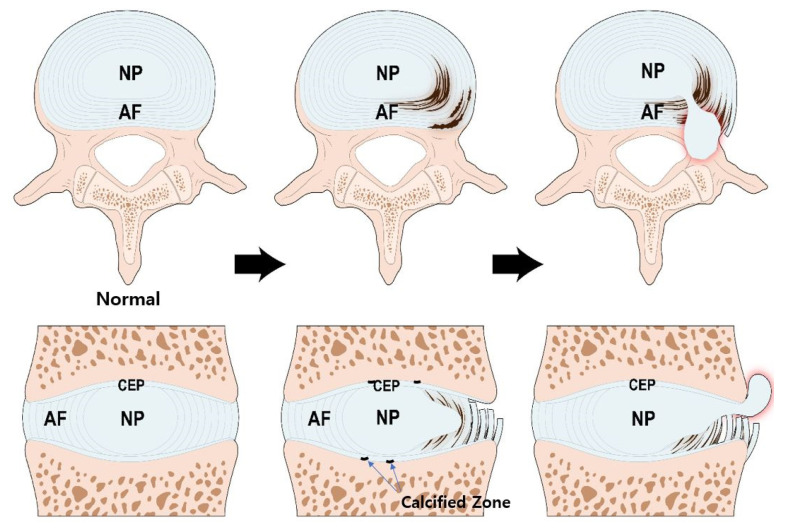
Structure of the IVD and pathogenesis of IVD degeneration. During the aging process, calcification occurs in the bone-cartilage junction of CEP. The calcified zone is impermeable thereby, reducing nutrient transport and inhibiting IVD cell metabolism. The NP cells, originally under adverse condition, are the first to be affected. Altered aggrecan composition and reduced type II collagen weakens the structural integrity of NP. Myxomatous degeneration in AF can disrupt its highly organized collagen fiber arrangement. These processes increase the potential IVD structural damage. IVD; intervertebral disc, NP; nucleus pulposus, AF; annulus fibrosus, CEP; cartilage endplate.

IVD is a complex joint structure comprising three main components. NP is the center of the IVD and is gelatinous and resilient. The AF circumferentially encapsulates the NP while the CEPs are located above and below the NP and AF (Figure 1).

NP comprises proteoglycan, type II collagen fibers, and elastin fibers [37,38]. The proteoglycan of the NP largely comprises aggrecan, which has a high level of anionic glycosaminoglycan (GAG). Aggrecan is composed of a long core protein found at its center, and approximately 100 chondroitin sulfate and 30 keratan sulfate chains bound covalently to the core protein [39]. Keratan sulfate, constituting the aggrecan of NP, is longer than that in articular cartilage tissue, but is considerably shorter than chondroitin sulfate [40]. The core protein has a globular domain that can bind to hyaluronan. Thus, hundreds of aggrecans combine with hyaluronan to form large proteoglycan aggregates [40]. These proteoglycan aggregates are responsible for the hydrophilic nature of the nucleus. The rich GAG chains enable the NP to have osmotic properties and allow the retention of fluid required to maintain NP height and turgor against compressive loads.

AF is a heterogeneous fibrocartilaginous tissue and has been shown to consist of outer and inner AF [41]. The outer AF comprises a series of concentrically arranged lamellae, each formed by parallel bundles of type I collagen fibers. In each layer, collagen fibers are oriented approximately 60° to the vertical axis of the spine and are arranged parallel to each other. Between adjacent lamellae, the collagen fibers are arranged perpendicularly to provide maximal tensile strength [38]. The inner AF serves as a transition between the outer AF and NP. The inner AF has a higher type II collagen and proteoglycan content within its interlamellar matrix than that in the outer AF [42,43]. Fibers of the outer AF are firmly anchored into the vertebral bodies, whereas the inner fibers are interconnected with the CEP [34].

NP and AF act synergistically during normal activities. The NP can withstand axial compressive loads due to its intrinsic hydrostatic pressure, whereas the AF can resist heavy tensile stresses [4]. When the disc is compressed, hydrostatic pressure is generated within the NP. The NP expands radially to engage the AF, wherein tensile strength further resists expansion [42,44].

The CEP is a thin plate of hyaline-like cartilage (0.2~0.8 mm) that primarily comprises proteoglycan and type II collagen [6]. The collagen fibers of the CEP are mainly aligned parallel to the vertebral body and are connected to the collagen fibers of the inner AF. However, the integration between collagen fibers in the NP and CEP is more convoluted [45]. The CEP is not anchored into the bony endplate; thus it can be readily separated from the vertebral body when a shearing force is applied in a traumatic situation [5]. The endplate distributes intradiscal pressures over the surface of the adjacent vertebral body and prevents pressurized NP from bulging into the vertebral body.

IVD is the largest avascular tissue in the human body. The blood vessels surrounding the IVD during the early phases of development subsequently disappear. The outer AF receives direct blood supply; however, the supply is confined to the periphery. The NP and inner AF are supplied by diffusion through the CEP. The capillary network which arises from the vertebral arteries, penetrates the subchondral bone, and terminates in loops at the bone and CEP junction [15]. The diffusivities are significantly associated with matrix porosity. The porosity of the CEP is significantly higher in the central region adjacent to the NP [46].

Diffusion through the CEP is the main nutrient supply pathway to IVD [47,48,49]. Small molecules, such as glucose and oxygen, leave vertebral capillaries and are diffused through the CEP, they then reach the cells of NP and AF. A concentration gradient for diffusion is established by maintaining a balance between the rate of blood supply and the rate of cellular consumption. Waste products, such as lactic acid, are removed by the reverse pathway [50].

## 3. Pathogenesis of IVD Degeneration

IVD degeneration is a progressive cascade of cellular, compositional, and structural changes [38,51]. Since IVD is an avascular tissue, glycolysis is the sole supply of cellular energy, which uses glucose and generates lactic acid [52]. IVD cells use less oxygen compared with other cells, consequently producing less carbon dioxide [53]; however, they require oxygen for function [54]. The viability of the disc cell is maintained when glucose is sufficient and lactic acid is regulated below a certain level [50]. In acidic environment, the rate of glycolysis and oxygen uptake decrease even when glucose levels are maintained, thus cell death increases [55].

The rates of matrix synthesis and degradation of disc cells are influenced by the local extracellular oxygen and pH levels [50]. The matrix synthesis rate is the highest at 5% oxygen, and if the oxygen tension falls below 5%, the synthesis rate is significantly reduced to the oxygen-tension dependent manner. At low oxygen tension, the production of macromolecules, such as sulfated GAG and protein, is greatly inhibited. Matrix component synthesis is higher at pH 7.0, than at pH 7.4; furthermore, when pH is decreased below 7.0, the synthesis rate drop steeply [56]. Matrix degradation is less sensitive to pH; the activities of metalloproteinase, a matrix catabolic enzyme, are similar at pH 7.0 and 6.4 [57]. Therefore, an acidic pH increase the matrix breakdown rate by inhibiting synthesis [50].

During the aging process, calcification occurs in the bone-cartilage interface of the endplate [58], which is impermeable, preventing diffusion, reducing nutrient transport, and inhibiting disc cell metabolism [59]. The CEP tissue of degenerated IVD contains high level of calcium ions, which lowers the secretion and accumulation of type I, II collagen and proteoglycan and reduces the CEP permeability [59].

The NP, the center of the disc, has the lowest concentration of glucose and oxygen, and the highest concentration of lactic acid [52]. The activity and viability of NP cells at the center of the disk, originally under adverse conditions, are the first to be affected [60]. Early degenerative changes in the NP involve an increase in the proteolytic degradation of aggrecan, and an increase in non-aggregating proteoglycan. The accumulation of degraded proteoglycan hinders the diffusion of glucose and oxygen. The proportion of GAG chains of aggrecan also changes; chondroitin sulfates, which are longer and more negatively charged, are decreased, and heparan sulfate and keratan sulfate, which are shorter and less negatively charged, are increased. These changes reduce the viscosity and hydrostatic pressure of NP. Thus, the ability of the NP to resist axial loading is decreased, the height of the NP is decreased, and the NP become bulging [61].

Major collagens constituting the IVD are type I and type II collagen, which are distributed radially in opposing concentration gradients. Type I collagen predominantly comprises fiber bundles of the AF, whereas type II collagen is the principal component of the random fibrillar NP network [62]. The synthesis of type II collagen peaks during early life but decreases gradually as degeneration progresses. The number of collagen cross-links is decreased due to the reduction in type II collagen; therefore, the structural integrity of the NP is weakened [63].

Type I collagen replaces type II collagen in the NP, making it a more fibrotic tissue. When degeneration proceeds, myxomatous degeneration occurs in the AF, disrupting the highly organized collagen fiber arrangement of AF, and disorganizing the collagen and elastin networks. When the collagen network is damaged, the biomechanics of the disc are significantly altered and the potential for structural damage is increased [36].

## 4. Expression of HIF and Signal Transduction of HIF-1α in IVD

### 4.1. Expression Patterns of HIF-1α and HIF-2α in IVD

Since the IVD is an avascular structure, its disc supply depends mainly on diffusion. A low supply of nutrients causes a decrease in oxygen, affecting cell function and synthesis of the extracellular matrix [15,50].

The HIF family of proteins contains HIF-1, HIF-2, and HIF-3, comprising an α-subunit and a constitutively expressed β-subunit. Among the HIF family, HIF-1 and HIF-2 play a crucial role for the biologic regulation of NP cells.

HIF-1 is an essential transcription factor that regulates the survival and functioning of NP cells in the avascular niche of the IVD [34,64]. Richardson et al. [17] demonstrated the expression of HIF-1α in normal and degenerative IVDs, and HIF-1α was markedly expressed in NP cells of degenerative IVD. Through western blot and immunohistochemistry analysis, Rajpurohit et al. [24] revealed that the expression of HIF-1α was only observed in NP cells, but not in AF and CEP cells. Ha et al. [32] revealed that HIF-1α expression and apoptosis occur in herniated disc areas and observed a considerable correlation between the expression of HIF-1α and apoptosis.

HIF-2α of NP cells has been reported in rats and its transactivation significantly increased under hypoxia [21]. Huang et al. suggested that HIF-2α is a catabolic regulator during disc degeneration, which its suppression decelerate ECM degradation and hence it may be a therapeutic target against IVD degeneration [65].

### 4.2. Signal Transduction Pathway of HIF-1α in IVD

**Figure 2:**
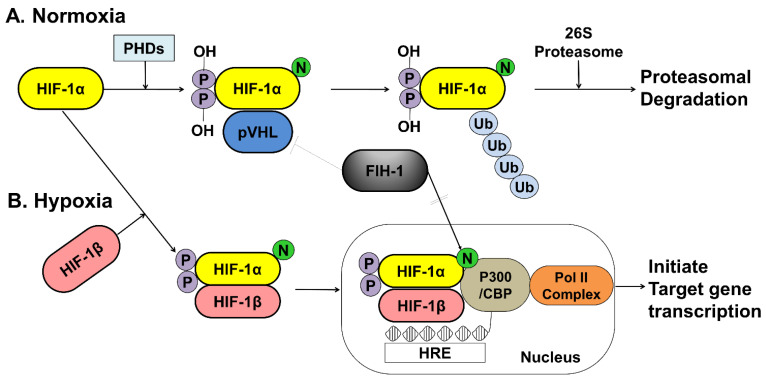
A schematic representation of the oxygen-dependent regulation of HIF-1α under normoxic and hypoxic conditions. (**A**) Under normoxic conditions, HIF-1α is hydroxylated at specific proline residues by PHD enzymes on the proline residues, which leads to polyubiquitination and proteasomal degradation of HIF-1α by the VHL protein, a component of E3 ubiquitin-protein ligase. (**B**) Under hypoxic conditions, PHD enzyme activity is inhibited, thereby allowing HIF-1α to accumulate and translocate to the nucleus, where it dimerizes with HIF-1β and binds to the HRE sequences of target gene promoters. The FIH-1, an asparagine hydroxylase, regulates the transcriptional activity of HIF-1 binding to VHL and represses HIF-1 transactivation by preventing the binding of the transcriptional coactivator p300/CBP to the HIF-1α. HIF; hypoxia inducible factor, PHDs; prolyl hydroxylase domain proteins, pVHL; protein von Hippel Lindau, CBP; Creb-binding protein, HRE; hypoxia-response element, Pol II; DNA polymerase II complex, Ub; Ubiquitous, FIH-1; factor inhibiting HIF-1.

The activity of HIF-1α within a cell depends on oxygen sensitivity, which decrease in response to normoxia and increases under hypoxic conditions (Figure 2).

Under normoxic conditions, HIF-1α is hydroxylated at two specific proline residues (Pro402 and Pro564) in the oxygen-dependent degradation domain by the prolyl 4-hydroxylase domain-containing (PHD) enzyme, which leads to polyubiquitination and proteasomal degradation of HIF-1α by the von Hippel–Lindau tumor suppressor protein, a component of E3 ubiquitin-protein ligase [66,67]. Under hypoxic conditions, PHD enzyme activity is inhibited, and HIF-1α is spared from polyubiquitination and proteasomal degradation, thereby allowing HIF-1α to accumulate and translocate to the nucleus, where it dimerizes with HIF-1β and binds to the hypoxia-responsive element (HRE) sequences of target gene promoters. This protein complex, which binds to an enhancer, the HRE, in HIF-a target genes, initiates gene transcription. Semenza et al. reported that the factor inhibiting HIF-1 (FIH-1), which is an asparagine hydroxylase, and regulates the transcriptional activity of HIF-1, which also demonstrates that FIH-1 binds to VHL [68]. The FIH-1 hydroxylation of Asn803 on HIF-1α represses HIF-1α transactivation by preventing the binding of the transcriptional coactivator p300/CBP to the HIF-1α C-transactivation domains [69,70] (Figure 2).

The Fas and Fas ligand (FasL) system transfers a death signal that rapidly commits cells to apoptosis and is expressed together in the disc of herniated IVD [71]. Fas can be expressed in various cell types, whereas FasL expression appears to be more restricted. FasL, a type II membrane protein of 36 kDa, belongs to the tumor necrosis factor family, and upon binding to the Fas, it acts as a cell-death-triggering ligand that induces apoptosis [72]. Zeng et al. [30] found that HIF-1α may induce the expression of Galectin-3(gal-3) and sequentially inhibit Fas/FasL-mediated apoptosis of NP cells and further confirmed that HIF-1α combined with gal-3 HRE that site-directed mutagenesis of HRE completely blocked hypoxic induction of gal-3 promoter activity.

## 5. Regeneration for IVD Degeneration—Focused on HIF-1α

### 5.1. Main Roles of HIF-1α in IVD Degeneration

#### 5.1.1. Promotion of Extracellular Matrix in NP Cells

**Figure 3:**
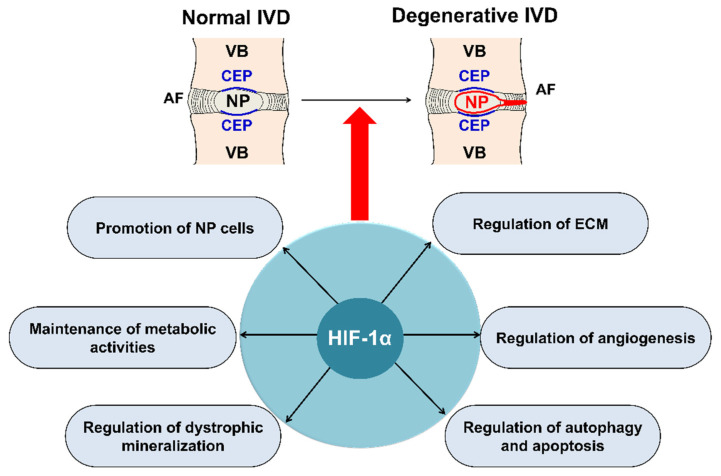
Main roles of HIF-1α in IVD degeneration. HIF-1α, involved in IVD degeneration, promotes ECM in NP, maintains the metabolic activities of NP, regulates dystrophic mineralization of NP, and regulates angiogenesis, autophagy, and apoptosis during IVD degeneration. HIF; hypoxia inducible factor, IVD; intervertebral disc, ECM; extracellular matrix, NP; nucleus pulposus, AF; annulus fibrosus, CEP; cartilage endplate, VB; vertebral body.

Regulation of human NP survival and ECM synthesis are therapeutic strategies for IVD degeneration [14] (Figure 3). In the NP of the disc, ECM is predominantly composed of proteoglycans and type II collagen [12]. Proteoglycans are abundant in the NP, which permits the IVD to resist compressive loads for spinal stability and allows slight movement of the spine [22]. Recently, biological studies on the prevention of early disc degeneration by promoting ECM synthesis, including NP cells, by upregulating HIF-1α have been reported [73,74,75,76,77,78]. Wang et al. [79] revealed that the high expression of long non-coding RNA(RP11-296A18.3) promotes HIF-1α expression through low expression of mitochondrial RNA (miR-138), thus promoting NP cell proliferation and ECM synthesis. Chen et al. [76] revealed that the PHD/HIF-1/CA12 pathway may affect disc degeneration by regulating ECM anabolism, and this pathway activity could be a valuable therapeutic approach to IVD degeneration.

The receptors, ligands, and target genes of the NOTCH1 signaling pathway are expressed in IVD cells, and NOTCH1 signaling is crucial for the maintenance of NP cell proliferation under hypoxic conditions of the IVD [80]. HIF-1α increases the expression of type II collagen and aggrecan in NP cells via the NOTCH1 pathway [81]. Further research on ECM synthesis in NP cells by promoting HIF-1α, which is the most crucial mechanism, is warranted.

#### 5.1.2. Maintenance of the Metabolic Activity of NP Cells

HIF plays a significant role in the preservation of the metabolic activities of NP cells in IVD [23,24,82] (Figure 3). Hypoxia-responsive glucose transporter (GLUT), located on the cell membrane, is an important gene for promoting anaerobic glycolysis of NP cells in IVD [31,83]. Richardson et al. [17] showed that HIF-1α, GLUT-1, GLUT-3, and GLUT-9 were co-expressed in normal human IVDs, and an increase in HIF-1α expression was associated with an increase in the expression of GLUT-1, GLUT-3, and GLUT-9 in NP cells; however, this association has not been observed in AF cells. Interestingly, they also observed that the expression of GLUTs increased as IVD degeneration progressed. HIF-1 maintains the metabolic activities of NP cells under a hypoxic environment in IVDs, mainly by regulating the expression of GLUT-1, GLUT-3, and GLUT-9.

HIF-1α also regulates mitochondrial energy metabolism [83,84]. Papandreou et al. [84] showed that HIF-1α stimulates glycolysis and actively inhibits mitochondrial function and oxygen consumption by inducing pyruvate dehydrogenase kinase 1.

#### 5.1.3. Regulation of Dystrophic Mineralization in NP Cells

Dystrophic mineralization due to aging and injury is a common problem observed in soft tissues and can cause several diseases, including calcification of cartilage, resulting in osteoarthritis [85,86]. The CEP calcification associated with the severity of IVD degeneration [87] causes the permeability of CEP to decline, and nutrients and metabolic waste in the IVD may cause the occurrence and progression of IVD degeneration through an increase in HIF-1α [88]. Previous studies have shown that mutations in ankylosis protein homolog gene (ANK), a pyrophosphate transporter, could result in abnormal dystrophic mineralization in joints and bone [89,90] and the hypoxic status may influence ANK expression, which may be mediated by HIF-1α. HIF-1α controls the dystrophic mineralization of NP cells by suppressing ANK expression [29]. Dystrophic mineralization caused by abnormal expression of ANK can cause the IVD degeneration [29,91]. Skubutyte et al. [29] found that ANK was expressed in calcified areas of the CEP, and HIF-1α directly downregulate ANK expression, indicating that expression of HIF-1α can maintain the physiological level of ANK under physiological conditions, thereby preventing the occurrence of dystrophic mineralization and promoting NP cells to adapt to the hypoxic environment.

#### 5.1.4. Regulation of Angiogenesis during IVD Degeneration

Vascular endothelial growth factor (VEGF) and its receptors (VEGFRs) play important roles in both physiological and pathological angiogenesis [92]. Han et al. [93] demonstrated that the VEGF gene may increase the risk for the development of IVD degeneration and is also positively related to the severity of IVD degeneration. Vascularized IVD cells improve blood circulation and the nutrition of NP cells and play an important role in the spontaneous absorption of herniated IVD [94,95]. Kwon et al. [96] demonstrated that VEGF expression significantly activates HIF-1α under hypoxia during an inflammatory reaction. Agrawal et al. [21] demonstrated that HIF-1α may enhance NP cell survival in a specialized microenvironment of the IVD via HIF-1α-mediated VEGF expression. Wu et al. [97] indicated that the HIF-1α/VEGF signaling pathway plays a crucial role in the survival of NP cells and maintenance of the ECM balance in the development of IVD degeneration.

#### 5.1.5. Autophagy and Apoptosis during IVD Degeneration

Autophagy, the process of non-apoptotic, programmed cell death, is a physiological response to hypoxia and exhibits a complicated association with hypoxia [98,99]. The level of autophagy changes according to IVD degeneration and age [98,100]. It has been shown that NP cells show higher autophagy activity under normoxia than under hypoxia [98,101]. Hypoxic conditions facilitate NP cell survival via the downregulation of excessive autophagy, by restricting the production of reactive oxygen species and inactivating the AMPK/mTOR signaling pathway, which may be regulated by HIF-1α [101].

Excessive apoptosis and senescence of NP cells may lead to degeneration of the ECM; hence, HIF-1α could be a therapeutic target against IVD degeneration [102]. Using surgical IVD specimens, Gruber et al. [103] demonstrated that a high incidence of apoptosis was detected in IVD. HIF-1α is positively associated with NP cell apoptosis in herniated discs [32] and enhances the ability of NP cells to adapt to hypoxia and survive under hypoxic conditions.

### 5.2. HIF-1α Development Strategies for IVD Regeneration

Among the members of the HIF family, HIF-1β is a stable constitutively expressed protein in the nucleus and is not affected by the hypoxic state. However, the HIF-1α subunit has a short half-life of less than 5 min because under hypoxic conditions, PHD may be deactivated and cause degradation of HIF-1α. HIF-1α is the most important molecule that regulates the IVD degeneration [104].

Transplantation of NP cells into the IVD may be an effective treatment for degeneration. Wang et al. [105] demonstrated that the antiapoptotic effect and migratory capacity of transplanted NP cells increased the expression of ECM, promoted NP cell migration to the damaged area, and alleviated IVD degeneration. The expression of HIF-1α, which is enhanced under hypoxia, can promote cell proliferation, and maintain the functional phenotype of NP cells. Therefore, it is important to isolate and amplify NP cells during hypoxia for cell transplantation, which depends on the HIF-1α/GLUT 1 pathway [106].

Even under normoxic conditions, constitutively active HIF-1α can be obtained by point mutation or deletion and may be therapeutic a target for IVD degeneration. Previous studies have focused on the regulation of non-coding RNA that are associated with HIF-1α in IVD [79,104,105,107]. HIF-1α may also be a therapeutic target for non-coding RNAs regulating IVD degeneration. Liu et al. [104] reviewed the role of HIF-1α and suggested that HIF-1α and EMC can be used the evaluation criteria for the pathological diagnosis of the “internal grading” of the degree of microenvironmental disorders in IVD degeneration. Thus, HIF-1α may be a diagnostic tool for early IVD degeneration and can act as a therapeutic target for inhibiting them.

## 6. Conclusions

HIF-1α in NP cells represents a potential early diagnostic marker and therapeutic target against IVD degeneration. Development of diagnostic and therapeutic strategies targeting HIF-1α to promote ECM and regulate dystrophic mineralization in NP cells, maintain metabolic activity of NP cells, and regulate angiogenesis, autophagy, and apoptosis during IVD may be an effective treatment strategy for the prevention of IVD degeneration. Further studies on HIF-1α regulated pathways in discs may be valuable for early diagnosis, prevention, and treatment of IVD degeneration.

## Data Availability

Not applicable.

## Abbreviations

IVD	intervertebral disc
ATP	adenosine triphosphate
HIF-1α	hypoxia-inducible factor-1 alpha
NP	nucleus pulposus
AF	annulus fibrosus
CEP	cartilaginous end plate
GAG	anionic glycosaminoglycan
PHD	prolyl 4-hydroxylase domain-containing
VHL	von Hippel–Lindau tumor suppressor
HRE	hypoxia responsive element
FIH-1	factor inhibiting HIF-1
C-TAD	C-transactivation domains
FasL	Fas ligand
ECM	extracellular matrix
GLUT	glucose transporter
ANK	ankylosis protein homolog gene
VEGF	vascular endothelial growth factor
VEGFR	vascular endothelial growth factor receptor
AMPK	adenosine 5′-monophosphate-activated protein kinase
mTOR	mechanistic target of rapamycin
